# Predictors of Efavirenz Plasma Exposure, Auto-Induction Profile, and Effect of Pharmacogenetic Variations among HIV-Infected Children in Ethiopia: A Prospective Cohort Study

**DOI:** 10.3390/jpm11121303

**Published:** 2021-12-05

**Authors:** Adugna Chala, Birkneh Tilahun Tadesse, Tolossa Eticha Chaka, Jackson Mukonzo, Eliford Ngaimisi Kitabi, Sintayehu Tadesse, Anton Pohanka, Eyasu Makonnen, Eleni Aklillu

**Affiliations:** 1 Department of Pharmacology and Clinical Pharmacy, College of Health Sciences, Addis Ababa University, Addis Ababa 9086, Ethiopia; adugnadema@gmail.com (A.C.); eyasumakonnen@yahoo.com (E.M.); 2 Division of Clinical Pharmacology, Department of Laboratory Medicine, Karolinska Institutet, Karolinska University Hospital Huddinge, 141 86 Stockholm, Sweden; birknehtilahun@gmail.com (B.T.T.); anton.pohanka@sll.se (A.P.); 3 Department of Pediatrics, College of Medicine and Health Sciences, Hawassa University, Hawassa 1560, Ethiopia; ssintayehu2010@gmail.com; 4 Department of Pediatrics and Child Health, Adama Hospital Medical College, Adama P.O. Box 84, Ethiopia; tecb2006@gmail.com; 5 Department of Pharmacology and Therapeutics, College of Health Sciences, Makerere University, Kampala P.O. Box 7072, Uganda; mukojack@yahoo.co.uk; 6 Division of Pharmacometrics, Food and Drugs Administration, Office of Clinical Pharmacology, Silver Spring, MD 20993, USA; engaimisi@gmail.com; 7 Center for Innovative Drug Development and Therapeutic Trials for Africa, College of Health Sciences, Addis Ababa University, Addis Ababa 9086, Ethiopia

**Keywords:** efavirenz, CYP2B6, autoinduction, pharmacogenetics, children

## Abstract

(1) Background: Efavirenz plasma concentration displays wide between-patient variability partly due to pharmacogenetic variation and autoinduction. Pediatric data on efavirenz pharmacokinetics and the relevance of pharmacogenetic variation are scarce, particularly from sub-Saharan Africa, where >90% of HIV-infected children live and population genetic diversity is extensive. We prospectively investigated the short- and long-term effects of efavirenz auto-induction on plasma drug exposure and the influence of pharmacogenetics among HIV-infected Ethiopian children. (2) Method: Treatment-naïve HIV-infected children aged 3–16 years old (*n* = 111) were enrolled prospectively to initiate efavirenz-based combination antiretroviral therapy (cART). Plasma efavirenz concentrations were quantified at 4, 8, 12, 24, and 48 weeks of cART. Genotyping for *CYP2B6, CYP3A5, UGT2B7, ABCB1,* and *SLCO1B1* common functional variant alleles was performed. (3) Results: The efavirenz plasma concentration reached a peak at two months, declined by the 3rd month, and stabilized thereafter, with no significant difference in geometric mean over time. On average, one-fourth of the children had plasma efavirenz concentrations ≥4 µg/mL. On multivariate analysis, CYP2B6*6 and *ABCB1c.3435 C* > *T* genotypes and low pre-treatment low-density lipoprotein (LDL) were significantly associated with higher plasma efavirenz concentration regardless of treatment duration. Duration of cART, sex, age, nutritional status, weight, and *SLCO1B, CYP3A5, UGT2B7,* and *ABCB*1 rs3842 genotypes were not significant predictors of efavirenz plasma exposure. (4) Conclusion: Pre-treatment LDL cholesterol and CYP2B6*6 and *ABCB1c.3435 C > T* genotypes predict efavirenz plasma exposure among HIV-infected children, but treatment-duration-dependent changes in plasma efavirenz exposure due to auto-induction are not statistically significant.

## 1. Introduction

HIV infections remain a global public health problem, particularly in sub-Saharan Africa (SSA). According to the 2020 UNAIDS estimates, around two-thirds of the 1.7 million HIV-infected children (0–14 years) worldwide live in SSA [1]. Eastern and southern Africa are the most affected regions with a high prevalence of infected children. In Ethiopia, the estimated total number of children living with HIV in 2020 was around 44,000 [1]. Combination antiretroviral therapy (cART) has reduced AIDS-associated morbidity and mortality and improved the quality of lives of HIV-infected adults and children across the world. The global trend has shown an increased number of adults and children living with HIV, primarily due to a faster decline of AIDS-related mortality than new HIV infections due to sustained access to antiretroviral therapy. To achieve Sustainable Development Goal 3, UNAIDS adopted a strategy to end HIV/AIDS as a public health threat by 2030, setting ambitious targets to be achieved progressively [2]. However, children were far from the UNAIDS 90-90-90 goals, were not getting optimum treatment, and had lower rates of viral suppression than adults [1,3,4]. Recently we reported poor viral suppression [5,6], high rates of viral drug resistance [7,8], and treatment-associated adverse events [9,10] among HIV-infected Ethiopian children.

Efavirenz has served as the preferred first-line antiretroviral drug for more than a decade. Currently, the World Health Organization (WHO) recommends dolutegravir as the preferred first-line regimen for adults and children to whom dosing recommendation is available [4]. However, the efavirenz-based regimen is still the preferred first-line for children ≥3 years old in many African countries, including Ethiopia, as switching needs time [11]. Efavirenz is metabolized in the liver by genetically polymorphic cytochrome P450 enzymes primarily by CYP2B6 and to a lesser extent by CYP3A4, CYP3A5, CYP2A6, CYP1A2, and UGT2B7 [12]. Efavirenz induces multiple CYP enzymes and transporter proteins, including p-glycoproteins and organic-anion-transporting polypeptides (OATPs) [13]. Both p-glycoproteins and OATPs transport numerous drugs, including antiretrovirals. Although in vitro studies have reported that P-glycoprotein and OATP1B1 may not be the main cellular transporter proteins for efavirenz [14], the significant impact of the *ABCB1* genotype on plasma efavirenz concentrations in healthy volunteers [15] and HIV patients has been reported [16,17,18].

Efavirenz plasma concentration displays wide variations between patients and different populations, partly due to pharmacogenetic variations [18,19]. The pharmacokinetics, pharmacogenetics, and auto-induction profiles of efavirenz are well characterized in the adult population. Based on population pharmacokinetic modeling prediction, several studies, including ours, recommended a lower efavirenz dose than the standard (600 mg/day) dose for adult patients [20,21,22], which was later supported by findings from randomized clinical trials [23,24]. Consequently, low dose (400 mg/day) efavirenz was adopted by the WHO as an alternative first-line drug for adults [25]. It is common practice that most antiretroviral doses are approved for pediatric use based on pharmacokinetics and pharmacogenetic data extrapolated from adult studies [26]. However, due to lack of supporting data, there is no specific guidance on the use of low-dose efavirenz for children.

Overall pediatric data on efavirenz pharmacokinetics and the relevance of pharmacogenetic variation is scarce, mainly from sub-Saharan Africa, where >90% of HIV-infected children globally live and population genetic diversity is extensive [26,27]. The few available studies have reported wide between-patient variability in plasma efavirenz concentration among black African children infected with HIV [28,29,30]. The pharmacokinetics and pharmacogenetics of efavirenz and its association with treatment outcome have been well investigated among adult patients in Ethiopia [31,32,33,34]. This prospective cohort study investigated factors influencing the short- and long-term efavirenz plasma exposure and autoinduction profile among HIV-infected children in Ethiopia.

## 2. Materials and Methods

### 2.1. Study Design, Area, and Participants

This multi-centered prospective pharmacokinetic and pharmacogenetic study was conducted between April 2017 and August 2019. A total of 111 ART-naïve HIV-infected children aged from 3 to 16 years were prospectively recruited and enrolled at seven HIV/AIDS treatment centers located in two adjacent administrative regions in Ethiopia: Oromia and Southern Nations Nationalities and Peoples Region (SNNPR), which have diverse ethnic groups. In the Oromia region, the study centers were Adama Hospital, Asela Referral and Teaching Hospital, and Shashemene General Hospital. The study sites in SNNPR region were Hawassa University Comprehensive Specialized Hospital, Adare General Hospital, Hosana Referral Hospital, and Arbaminch General Hospital. These regions were selected because of high HIV prevalence and population diversity, which is generalizable to the nation.

### 2.2. Antiretroviral Therapy and Laboratory Analysis

At enrollment, complete medical history, clinical examinations, and laboratory tests including complete and differential blood counts, platelet counts, CD4 count, HIV viral load, and liver enzymes were performed. Subsequently, study participants initiated a first-line pediatric cART regimen containing efavirenz (EFV) and lamivudine (3TC) with either zidovudine (AZT), abacavir (ABC), or tenofovir (TDF) following the Ethiopian national pediatric HIV treatment guideline [11]. All children ≥3 years old who weighed ≥10 kg, and adolescents ≥10 years older who weighed <35 kg received weight-band dosing of EFV + 3TC + AZT or ABC as described in the national HIV treatment guideline [11]. Adolescents ≥10 years old who weighed ≥35 kg received an EFV 600 mg + TDF + 3TC fixed-dose combination once daily. Treatment adherence was assessed by parent/caregiver and child/adolescent interview at each study visit. The study participants were prospectively followed up to 48 weeks while on cART to monitor plasma efavirenz exposure. Whole blood samples at 12 to 16 h post efavirenz dose were collected on the 4th, 8th, 12th, 24th, and 48th weeks of starting ART. Plasma samples were prepared immediately, and aliquots were kept at −80 °C for analysis.

### 2.3. Genotyping for CYP2B6, CYP3A5, SLCO1B1, ABCB1, and UGT2B7

Genomic DNA was extracted from whole blood samples using a QIAamp DNA Blood Midi Kit (Spin Protocol) (Qiagen GmbH, Hilden, Germany). Genotyping for common functional variant alleles in five genes relevant for efavirenz disposition was conducted by real-time PCR using Taqman^®^ assay reagents for allelic discrimination (Applied Biosystems Genotyping Assay) as described previously [19,35]. Common functional variant alleles were selected for genotyping based on the reported impact on enzyme activity and relevance for the black-African population. Allelic discrimination PCRs were performed using TaqMan^®^ genotyping assays with the following ID numbers for each SNP: C__7817765_60 for CYP2B6*6 (c.516G>T, rs3745274), C__26201809_30 for CYP3A5*3 (c.6986A > G, rs776746), C__30203950_10 for CYP3A5*6 (rs10264272), C__32287188_10 for CYP3A5*7 (g.27131_27132insT rs41303343), C__11711730_20 for *ABCB1* (c.4036A > G rs3842), C__7586657_20 for *ABCB1* (c.3435C > T rs1045642), C__30720663_20 for *UGT2B7* (372G > A rs7662029), C__1901697_20 for SLCO1B1*1B (c.388A > G rs2306283), C__30633906_10 for SLCO1B1*5 (c.521T > C rs4149056), and C__1901709_20 for *SLCO1B1 (g.38664C > T* rs4149032). Genotyping was performed using a QuantStudio 12K Flex Real-Time PCR system (Life Technologies Holding, Singapore, Singapore). The final volume for each reaction was 10 µL, consisting of 2X TaqMan^®^ fast advanced master mix (Applied Biosystems, Waltham, MA, United States), TaqMan^®^ 20 X/40 X drug metabolism genotyping assay mix (Applied Biosystems), and genomic DNA. The PCR profile consisted of an initial step at 60 °C for 1 min, a hold stage at 95 °C for 20 s, and 40 cycles (step 1 with 95 °C for 3 s, step 2 with 60 °C for 30 s), and then a read stage with 60 °C for 30 s.

### 2.4. Quantification of Efavirenz Plasma Concentration

#### 2.4.1. Chemicals and Reagents

Efavirenz reference standard was purchased from Merck (Darmstadt, Germany). Deuterated-efavirenz (rac Efavirenz-d4) was purchased from Toronto Research Chemicals (Toronto, ON, Canada). Acetone, acetonitrile, ammonium acetate, isopropanol, methanol, and acetic acid of mass spectrometry (MS) grade were purchased from Merck (Darmstadt, Germany). Blank human plasma was obtained from the blood bank of the Karolinska University Hospital Huddinge (Stockholm, Sweden).

#### 2.4.2. LC-MS/MS Method and Validation

An LC-MS/MS system consisting of an Acquity Ultra Performance LC-system coupled to a Xevo TQ-S Micro (Waters, Milford, MA, USA) was used to quantify plasma efavirenz concentration. The MS/MS was operated in positive ionization mode. The data were processed using MassLynx 4.2 (Waters). In brief, plasma calibration samples were prepared by spiking blank plasma samples with efavirenz and were included in each analytical run. Quality control samples were also prepared by spiking plasma blanks to obtain low and high concentrations. The lower and upper limits of quantification were 15.8 and 15,800 ng/mL, respectively.

Plasma efavirenz concentration was determined by direct injection of the supernatant after protein precipitation using acetonitrile. In brief, a 50 µL aliquot of plasma samples was added into 200 µL acetonitrile solution containing internal standard (500 ng/mL of efavirenz-d4 in methanol), vortexed for 30 s followed by centrifugation for 5 min at 2100× *g*. An amount of 5 µL of the supernatant was injected onto the LC-MS/MS system. The chromatographic system consisted of an Acquity UPLC BEH C18 reversed-phase column 2.1 × 50 mm, 1.8 μm with 0.1% aqueous formic acid as mobile phase A and MeOH as mobile phase B. The initial composition of the mobile phase was 50% B, followed by a linear gradient to 99% in 1.5 min, with a flow rate of 0.4 mL/min. Total analysis/run time for each sample was approximately 3 min.

Efavirenz was monitored by the transition m/z 316 > 168 and the efavirenz-d4 by 320 > 172. The calibration curve was constructed by linear regression of the analyte/internal standard area ratios with an applied weighing of 1/x2. Accuracy was within ±10% throughout the quantification range. Precision was below 6 CV% except for LLOQ (below 10 CV%). The method was validated according to the European Medicines Agency Guideline on bioanalytical method validation [36].

### 2.5. Statistical Data Analysis

Study participants’ sociodemographic and baseline clinical parameters are summarized as the median and interquartile range (IQR) or as frequency and percentages. For assessment of the nutritional status of the children, anthropometric measurements were converted to height for age Z score (HAZ) and weight for age Z score (WAZ) using WHO Anthro-Plus software version 1.0.4. Body mass index (BMI) for age percentiles was calculated using the age-and-sex-specific percentile for BMI as per the Centers for Disease Control calculator for children and teens aged 2–19 years.

Efavirenz plasma concentrations were transformed to log10 values before statistical analysis. The dataset was reshaped from a wide to a long format in which each individual subject had five records or less for weeks 4, 8, 12, 24, and 48. One-way analysis of variance (ANOVA) was used to identify genotype association with efavirenz plasma concentration measured at different weeks of ART. Change in efavirenz concentration over time was analyzed using a generalized linear mixed-effect model (LME). Univariate followed by multivariate analyses using LME models were conducted to determine predictors of efavirenz concentrations and to quantify the unexplained between- and within-subject variations in efavirenz exposure at different weeks of treatment. The LME modeling estimated fixed effect (predictor coefficients) and random effect parameters (between and within-subject variabilities in efavirenz concentrations). The LME models were therefore formulated as follows.

$\mathrm{EFV}|=|\alpha_{i}\times {\mathrm{Week}}_{i}|+\beta_{j}\times {\mathrm{Predictor}}_{j}|+\epsilon$, where i is a vector of weeks on treatment, in this case, these are 4, 8, 12, 24, and 48; ϵ represents random-effect parameters; j represents additional predictors that were separately added to the model for individual evaluation. The LME model was developed in 3 steps. In the first step, time on treatment (weeks) was used as the only predictor in the model. In the second step, univariate models were developed, which included time on treatment plus one additional predictor. In the last step, all additional predictors that were physiologically plausible, non-collinear, and with *p*-values ≤ 0.2 were included in the final LME models.

Descriptive statistics analysis for baseline characteristics was performed using Statistical Package for Social Sciences (SPSS) software, version 20.0 (IBM Corporation, Somers, NY, USA). The R software package (The R Foundation for Statistical Computing, Vienna, Austria) and GraphPad Prism version 7 (version 7, San Diego, CA, USA) were used for the analysis and presentation of the results. *p* values < 0.05 were considered statistically significant.

### 2.6. Ethical Considerations

The study received ethical approval from the SNNPR Regional Health Bureau Institutional Review Board (IRB), Addis Ababa University College of Health Sciences (Protocol No: 053/16/Pharma), and the National Research Ethics Review Committee, Ministry of Science and Higher Education-Ethiopia (Ref NO 3.10/166/2016). Prior to giving consent, study participants and their parents/guardians received written and oral information in their local language about the study. Written informed consent from parents or legal guardians was obtained. For participants ≤12 years, written informed consent was obtained from their parent or guardian. For participants >12 years of age, informed consent was obtained from the parent or guardian, and assent was obtained from the study participant.

## 3. Results

A total of 111 newly diagnosed and ART-naïve HIV-infected children were enrolled prospectively to participate in this study, of whom 63 (56.8%) were boys. Thirty-nine (35.1%) children were orphans (having lost one parent or both). Eleven (9.9%) children did not complete the study due to lost to follow-up or transfer out from the study area.

### 3.1. Baseline Characteristics of Study Participants

The sociodemographic, clinical, and biochemical characteristics of the study participants are presented in Table 1. The median age of the children was nine years. The dose of efavirenz received per kilogram body weight per day ranged between 12 and 24 mg/Kg with a median dose of 17 mg/kg. Study participants’ body weight ranged between 8 and 49 Kg, with a median weight of 22 Kg. Following the WHO Z-score definition (<−2 ZS), 37.1% of the children were underweight; 33.9% were stunted, and 43.4% had a BMI for age percentile of less than the 5th percentile. More than half (60%) of the participants were in WHO clinical stages 2, 3, or 4. Most of the participants (53.2%) received a tenofovir TDF)/lamivudine/efavirenz regimen followed by abacavir/lamivudine/efavirenz (33%), and zidovudine/lamivudine/efavirenz regimen (13.6%).

### 3.2. Genotype and Allele Frequency Distribution

The variant alleles and respective genotype frequency distribution of *CYP2B6*, *CYP3A5*, *UGT2B7*, *ABCB1*, and *SLCO1B1* are presented in Table 2. There was no significant deviation between observed and expected genotype frequencies according to Hardy–Weinberg equilibrium. CYP3A5*7 (27131_27132insT) was absent. Haplotype analysis indicated no linkage disequilibrium between CYP3A4*1B (−392A > G), CYP3A5*3 (c.6986A > G), and CYP3A5*6 (c.14690G>A) [37,38]. Hence for haplotype–phenotype association analysis, study participants were grouped based on the number of functional CYP3A5*1 alleles (CYP3A5*1/*1 = two, heterozygous for *CYP3A5* *3 or *6 = one, homozygous for CYP3A5 *3 or *6 = zero).

### 3.3. Change in Plasma Efavirenz Concentration over Time

A total of 396 plasma samples were collected during the 48-week follow-up period. There was wide between-patient variability in plasma concentrations of efavirenz, and the coefficients of variation at the 4th, 8th, 12th, 24th, and 48th weeks of ART were 143%, 128%, 161%, 157%, and 142%, respectively. The proportions of children with sub-therapeutic (<1 µg/mL) plasma efavirenz concentration at the 4th, 8th, 12th, 24th, and 48th weeks of ART were 12.3%, 13%, 17.9%, 17.7%, and 12.3%, respectively. The proportions of children with supra-therapeutic (>4 µg/mL) plasma efavirenz concentration at the 4th, 8th, 12th, 24th, and 48th weeks of ART were 19%, 30%, 29%, 22%, and 29%, respectively.

**Figure 1:**
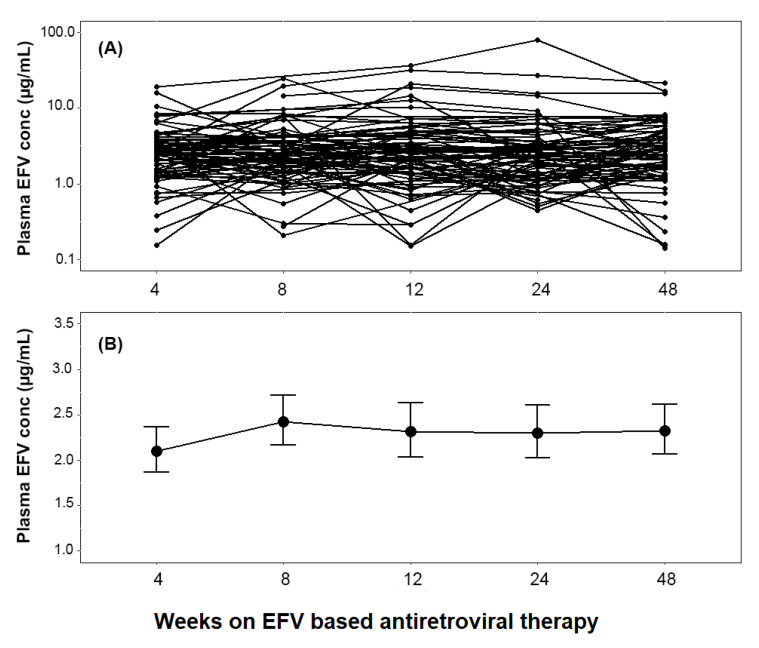
Change in plasma efavirenz (EFV) concentration over time among all children (**A** = line plot, **B** = mean ± standard error of mean).

The pattern and extent of change in plasma efavirenz concentration in each individual and the geometric mean at the 4th, 8th, 12th, 24th, and 48th weeks of ART are presented in Figure 1. Mixed effect model analysis indicated no significant within-subject variation in the geometric mean of plasma efavirenz concentrations over time (*p* = 0.89). Though not significant, the pattern of change in the mean log plasma efavirenz concentration over time displayed a moderate increase at the 8th week of ART, declined by the 12th week, and stabilized after that.

### 3.4. Effect of Genotype on Plasma Efavirenz Concentration at Each Study Time Point

**Figure 2:**
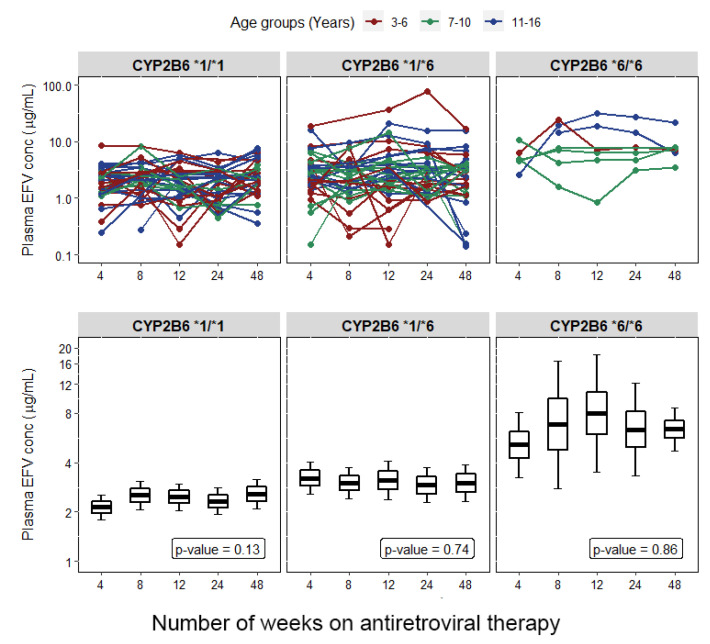
Change in plasma efavirenz (EFV) concentration over time among all children stratified by the *CYP2B6* genotype. Boxes represent mean ± se, whiskers are 95% confidence interval.

Association of genotype with plasma efavirenz concentration measured at the 4th, 8th 12th, 24th, and 48th weeks of efavirenz-based antiretroviral treatment among HIV-infected children using one-way analysis of variance is presented in Supplementary Table S1. Children with the CYP2B6*6/*6 genotype had significantly higher mean log efavirenz plasma concentration than homozygous wild-type (CYP2B6*1/*1) or heterozygous (CYP2B6*1/*6) at week 4 (*p* = 0.004, 0.09), week 8 (*p* = 0.002, <0.001), week 12 (*p* = 0.006, 0.37), week 24 (*p* = 0.002, 0.01), and week 48 (*p* = 0.01,0.01). The CYP2B6*6 genotype was significantly associated with therapeutic plasma efavirenz concentration categories (*p* < 0.001). There was no significant impact of *CYP3A5*, *UGT2B7*, *SLCO1B*1, and *ABCB1 c.4036A > G* genotypes on efavirenz plasma concentration at all study time points, except for *ABCB1 c.3435C > T*. Carriers of at least one *ABCB1 c.3435C > T* allele had significantly higher mean log efavirenz plasma concentration than those with the *ABCB1 c.3435 C/C* genotype at weeks 4 and 12 with *p*-values of 0.004 and 0.03, respectively. Although not statistically significant, children with at least one variant allele of *ABCB1 c.4036A > G* had a higher mean log efavirenz plasma concentration at all treatment weeks. The pattern and extent of change in the geometric mean of plasma efavirenz concentration at the 4th, 8th, 12th, 24th, and 48th weeks of ART among the different *CYP2B6* genotypes stratified by age group are presented in Figure 2.

### 3.5. Predictors of Plasma Efavirenz Exposure over Time

A univariate followed by a multivariate analysis was conducted to identify predictors of plasma exposure over time using the developed linear mixed effect (LME) models. The impact of sociodemographic, plausible clinical, and biochemical characteristics of the study participants presented in Table 1 were tested individually in a univariate analysis. In the following multivariate analysis, all predictors with *p*-values ≤ 0.2 from the univariate analysis were included in the model simultaneously, and the result is presented in Table 3. The unexplained between-subject and within-subject variability in efavirenz concentration was about 28% and 34%, respectively. CYP2B6*6 (*c.516G > T*), ABCB1 c. 3435 C > T genotype, and baseline LDL were significant predictors of efavirenz exposure regardless of weeks of treatment (Table 3). The coefficients of estimates were positive for CYP2B6*6 and *ABCB1 C3435T* genotypes, which means carriers of these variant alleles had a higher plasma concentration than those with a wild-type allele. However, the coefficient was negative for pre-treatment LDL concentration, indicating an association of increased LDL level with a decrease in plasma efavirenz exposure. Duration of antiretroviral treatment (in weeks) as a categorical predictor was included in the model and found to have no significant effect on the plasma efavirenz exposure.

### 3.6. Predictors of Efavirenz Concentration over Time among CYP2B6 *1/*1 Genotype

A subgroup analysis was performed to identify predictors of plasma efavirenz concentration over time among study participants with the CYP2B6*1/*1 genotype. In a univariate analysis, the impact of sociodemographic, potential pre-treatment clinical, and biochemical characteristics of the study participants presented in Table 1 were tested individually. In the following multivariate analysis, all predictors with *p*-values ≤ 0.2 from the univariate analysis were included in the LME model simultaneously to predict plasma exposure over time, and the results are presented in Table 4. Among children with the CYP2B6*1/*1 genotype, the unexplained between-subject and within-subject variability in efavirenz concentration was about 22% and 26%, respectively. The effect of duration of therapy on plasma efavirenz concentration was noted at week 8 of ART. While pre-treatment higher liver enzymes (ALT and ALP), total cholesterol, and triglycerides were positive predictors, LDL was a negative predictor of plasma efavirenz exposure regardless of weeks on ART among children homozygous for the functional CYP2B6*1 variant allele.

## 4. Discussion

Previous studies reported the association of pharmacogenetic variations and plasma efavirenz concentration with antiretroviral-treatment-induced neurotoxicity and liver enzyme abnormalities among HIV-infected patients [16,34]. Therefore, identifying predictors of efavirenz plasma exposure is essential to identify patients at risk of developing treatment-associated adverse events for personalized medicine. This study investigated the predictors of short- and long-term plasma efavirenz exposure among children on ART. The change in plasma efavirenz concentration over time was prospectively monitored as a surrogate marker to determine the time-course and extent of efavirenz autoinduction. Our main findings include (i) the presence of wide between-patient variability in efavirenz plasma exposure, but no significant time-dependent change over time among children. (ii) CYP2B6*6 (*c.516G > T*), *ABCB1 3435C > T* genotype, and low pre-treatment LDL concentration are significant predictors of high plasma efavirenz exposure regardless of the duration of therapy. (iii) Among the CYP2B6*1/*1 genotype, altered liver enzymes (ALT, ALP) and lipid profiles (total cholesterol, triglycerides, and LDL) influence plasma efavirenz exposure. (iv) There is no significant association of nutritional status (stunting and wasting), sex, age, *SLCO1B1, CYP3A5*, and *UGT2B7* genotype on plasma efavirenz concentration or autoinduction profile. To our knowledge, this is the first study to explore the short- and long-term efavirenz plasma exposure, and autoinduction profile among HIV-infected children using efavirenz plasma level as an index and to examine any implications of pharmacogenetic variations.

Efavirenz induces its own metabolism by increasing the expression of hepatic CYP2B6 enzyme via the human constitutive androstane receptor (hCAR) [39]. Currently, treatment of HIV is lifelong, and concomitant medication for the treatment of opportunistic infection is common. Therefore, characterization of the time course and magnitude of enzyme induction is essential to avoid possible drug interactions. Studies in primary human hepatocytes have indicated that efavirenz autoinduction is non-linear, and time- and concentration-dependent [39,40]. Using an endogenous marker, we previously monitored the time course and extents of CYP3A and CYP2B6 induction among adult HIV patients from Tanzania [41,42,43], Uganda [16], and Ethiopia [31,33,35]. Results from adult HIV-infected patients indicate that efavirenz autoinduction is long-term and significantly reduces its plasma concentration over time, particularly among the CYP2B6*1/*1 genotype. This contrasts with our current findings among children, where minor changes in plasma efavirenz concentrations over time were observed regardless of the *CYP2B6* genotype. Such inconsistent efavirenz autoinduction profiles between adults and children could be due to differences in age-related metabolic capacity or differences in administered dose. The observed non-significant effect of treatment duration on efavirenz plasma exposure over time may indicate that maximal induction reaches a “steady state” within the first four weeks of therapy at the given weight-band efavirenz dose for children. Nonetheless, the current efavirenz pediatric dosing based on the child bodyweight category results in no significant change on long-term plasma efavirenz exposure.

Notably, our study shows the existence of wide between-patient variability in efavirenz plasma concentration among children. In line with our previous reports from adult patients [18,19], the CYP2B6*6 genotype was significantly associated with plasma efavirenz concentration and therapeutic range categories among children. Although CYP2B6*6 allele frequency distribution is not significantly different between Ethiopian adults (29.7) [19] and children (30.1%), the proportion of patients in the various plasma efavirenz therapeutic range categories varied significantly between children and adult patients. The proportion of children with sub-therapeutic efavirenz plasma concentration levels (< 1 µg/mL) was much lower than adults [19]. However, the proportions of patients with plasma concentrations exceeding the safety threshold (≥4 µg/mL) were four-fold higher in children than adult patients [19]. The proportion of children with plasma efavirenz concentration ≥ 4 µg/mL at the 4th, 8th, 12th, 24th, and 48th weeks of ART were 19%, 30%, 29%, 22%, and 29%, respectively, while the proportion of adult patients with plasma efavirenz concentration ≥4 µg/mL at the 4th and 16th weeks of cART were 6% and 5%, respectively [19]. With the current recommended weight-band pediatric efavirenz dose, our findings indicate that on average one-fourth of Ethiopian HIV-infected children experience higher plasma drug exposure ≥4 µg/mL. This is a concern since such higher plasma efavirenz exposure is associated with treatment-induced neuropsychiatric manifestations and liver enzyme abnormalities [16,34], increased psychopathology [44], and poor long-term neurocognitive outcomes in HIV-infected children [45].

This study used linear mixed effect (LME) modeling to identify variables that predict plasma efavirenz concentration in children (Table 3 and Table 4). In line with previous reports [15,18,19], CYP2B6*6 (*c.516G > T*) and *ABCB1 c3435C > T* genotypes were significant predictors of higher efavirenz plasma concentration. CYP2B6 is the main enzyme responsible for the metabolism of efavirenz. *ABCB1* encodes P-glycoprotein, an ATP-dependent drug efflux pump, which is responsible for drug transport across extra- and intra-cellular membranes. Our findings reveal that sex, stunting, wasting, weight, and age had no significant role in predicting plasma efavirenz among children. The current pediatric HIV treatment guideline is only weight-based and does not consider enzyme maturation and genetic variations [46]. Hence, the current efavirenz dosing strategy for pediatric use might be inappropriate, especially for young children in sub-Saharan Africa, where the defective *CYP2B6* defective variant allele occurs at a higher frequency. Consideration of a pharmacogenetics algorithm might be more useful for determining the optimal dose of efavirenz for pediatric patients [20,47].

Both HIV infection and efavirenz-based ART alter lipid profiles [48,49]. Recently we reported a significantly higher prevalence of lipid profile abnormalities (hypertriglyceridemia and reduced HDL level) among HIV-infected children and adolescents who were on cART as compared to treatment-naïve HIV-infected children [9]. A cross-sectional study from South Africa reported the association between higher plasma efavirenz concentrations with fasting plasma lipid lipoproteins among HIV-infected adult patients [50]. The current study is the first prospective study to report the association of pre-treatment lipid profile with efavirenz pharmacokinetics. VLDL/LDL acts as a drug carrier and regulates the transport and metabolism of drugs in the body, and associated drugs are eliminated faster from the body by lipoprotein apheresis, a blood purification therapy that selectively removes VLDL/LDL particles from the bloodstream [51]. In line with this, we found a significant association between low LDL-cholesterol level at baseline (pre-treatment) with higher efavirenz plasma exposure regardless of the duration of therapy. Lack of a significant change in LDL level by ART [9] means that pre-treatment LDL level can serve as a reliable predictor of efavirenz plasma exposure, as observed in this study.

This study has some limitations. We could not utilize changes in intrinsic hepatic clearance of efavirenz to study the impact of efavirenz induction on its plasma exposures, as was done in previous studies among healthy adult subjects. Intensive pharmacokinetics sampling, which requires multiple blood withdrawals at each study time point over a one-year period from vulnerable patient populations (HIV-infected children) just for study purposes, was not possible due to ethical concerns. Likewise, we could not perform fasting lipid profile analysis for ethical reasons as children did not fast for the study purpose. Instead, we characterized the time course of induction and extent of changes in efavirenz plasma concentration profile in the same individual over time, whereby each child was effectively functioning as their own control, lowering the level of unexplained variance.

## 5. Conclusions

We report the presence of wide between-patient variability in plasma efavirenz exposure among HIV-infected children, partly due to *CYP2B6* and *ABCB1* pharmacogenetic variations. In addition, pre-treatment plasma LDL is a significant predictor of plasma efavirenz exposure. Duration of therapy-dependent changes in plasma efavirenz concentration due to autoinduction (as observed in adult patients) is not significant with the current weight-band efavirenz dosing among children. About one-fourth of the children had plasma concentrations exceeding supratherapeutic plasma efavirenz concentration (4 µg/mL), indicating that the current weight-band efavirenz dosing maybe unnecessarily high. Weight and age, which are the basis for the current pediatric efavirenz dosing, had no significant effect on plasma efavirenz concentration. For estimating the optimum pediatric efavirenz dose, *CYP2B6* genotype-based dose modifications may have more importance. Future pharmacokinetic and pharmacogenetic association studies with treatment outcome (efficacy and safety) measures are required to support a low dose efavirenz regimen among the pediatric population as confirmed in adult HIV patients.

## Figures and Tables

**Table 1:** Baseline demographic, clinical, and biochemical characteristics of study participants.

Categorical Variables		Proportion *n* (%)
Sex	Male	63 (56.8)
	Female	49(43.2)
Height for age Z-score	Normal	72 (66.1)
	Stunted	37 (33.9)
Weight for age Z-score	Normal	44 (62.9)
	Underweight	26 (37.1)
BMI for age percentile	5th–85th (Normal)	60 (56.6)
	<5th (Wasted)	46 (43.4)
Types of ART initiated	ABC/3TC/EFV	36 (33)
	AZT/3TC/EFV	15 (13.6)
	TDF/3TC/EFV	59 (53.6)
Hepatitis B virus surface antigen	Negative	106 (98.1)
	Positive	2 (1.9)
Hepatitis B virus antibody	Negative	105 (99.1)
	Positive	1 (0.9)
WHO clinical stage	Stage 1	44 (39.6)
	Stage 2	23 (20.7)
	Stage 3	33 (29.7)
	Stage 4	10 (9)
Any co-medication	Yes	13 (11.7)
	No	98 (88.3)
**Continuous Variables**		**Median (IQR)**
Age at enrolment (years)		9.0 (5–13)
Mid-upper arm circumference (cm)		15 (14–17)
CD4 count (cells/dL)		330 (200–671)
Viral load (copies/mL)		16,105 (1987–75,761)
Aspartate aminotransferase (units/L)		38 (30–48)
Alanine aminotransferase (units/L)		27 (20–39)
Alkaline phosphatase (units/L)		304 (188–410)
Blood urea nitrogen (mg/dL)		18 (12–27)
Total bilirubin (mg/dL)		0.8 (0.4–1.1)
Creatinine (mg/dL)		0.6 (0.4–0.7)
Albumin, median (mg/dl)		3.8 (3.1–4.2)
Hemoglobin (mg/dL)		12.4 (11.5–13.3)
Hematocrit (%)		37.4 (35.1–40.5)
Total cholesterol (mg/dL)		119 (95–150)
High-density lipoprotein (mg/dL)		47 (35–65)
Low-density lipoprotein (mg/dL)		45 (33–64)
Triglycerides (mg/dL)		106 (87–162)

IQR, interquartile range; BMI, body mass index; ART, antiretroviral therapy; TDF, tenofovir; 3TC, lamivudine; EFV, efavirenz; ABC, abacavir; AZT, zidovudine.

**Table 2:** Genotype and variant allele frequency distribution among HIV-infected children treated with efavirenz-based ART in Ethiopia.

Variant Allele	Minor Allele Frequency (%)	Genotype	Frequency (N)%
CYP2B6*6	30.1	*1/*1	49 (47.6)
		*1/*6	46 (44.6)
		*6/*6	8 (7.8)
CYP3A5*3	67.0	*1/*1	10 (9.7)
		*1/*3	48 (46.6)
		*3/*3	45 (43.7)
CYP3A5*6	10.2	*1/*1	82 (79.6)
		*1/*6	21 (20.4)
		*6/*6	0
*ABCB1 c.3435 C > T*	19.4	*C/C*	66 (64.1)
		*C/T*	34 (33)
		*T/T*	3 (2.9)
*ABCB1 c.4036A > G*	18.0	*A/A*	72 (69.9)
		*A/G*	25 (24.3)
		*G/G*	6 (5.8)
*SLCO1B1 g.38664 C >T*	52.9	*C/C*	25 (24.3)
		*C/T*	47 (45.6)
		*T/T*	31 (30.1)
SLCO1B1*5	13.6	*1/*1	78 (75.7)
		*1/*5	22 (21.4)
		*5/*5	3 (2.9)
SLCO1B1*1B	64.1	*A/A*	12 (11.7)
		*A/G*	50 (48.5)
		*G/G*	41 (39.8)
UGT2B7*2	45.6	*G/G*	27 (26.2)
		*A/G*	58 (56.3)
		*A/A*	18 (17.5)

**Table 3:** Predictors of plasma efavirenz exposure across weeks on antiretroviral treatment among all study participants.

Predictor	Predictor Values	Univariate *				Multivariate			
		Coefficient Estimates (Log10 Scale)	*p*	Between-Subject Variability	Within-Subject Variability	Coefficient Estimates (Log10 Scale)	*p*	Between-Subject Variability	Within-Subject Variability
Time on ART (Weeks)	Reference (Week 4)	0.335	0.000	34%	34%	0.181	0.17	28%	34%
	Week 8	0.052	0.34			0.057	0.31		
	Week 12	0.024	0.67			0.017	0.77		
	Week 24	−0.008	0.88			−0.009	0.88		
	Week 48	0.003	0.95			−0.006	0.91		
Type of ART regimen	Reference (ABC/3TC/EFV)	0.240	0.002	33%	34%				
	AZT/3TC/EFV	0.040	0.77			0.038	0.75		
	TDF/3TC/EFV	0.170	0.05			0.117	0.16		
Baseline LDL	Intercept	0.530	0.000	33%	34%				
	LDL	0.0001	0.02			−0.003	0.04		
Age at enrollment	Intercept	0.180	0.09	33%	34%				
	Age	0.020	0.10			0.009	0.36		
CYP2B6*6	Reference (*1/*1)	0.230	0.001	29%	34%				
	*1/*6 or *6/*6	0.610	<0.0001			0.574	0.00		
*ABCB1 c.3435 C > T*	Reference (C/C)	0.280	<0.0001	33%	34%				
	*C/T* or *T/T*	0.180	0.03			0.173	0.03		

* Only predictor variables with a *p*-value ≤ 0.2 from the univariate analysis that were included in the multivariate model are listed. LDL: low-density lipoprotein.

**Table 4:** Multivariate predictors of efavirenz exposure across weeks on treatment for subjects with CYP2B6*1/*1 genotype.

Predictor	Predictor Values	Univariate *				Multivariate			
		Coefficient Estimates (Log10 Scale)	*p*	Between-Subject Variability	Within-Subject Variability	Coefficient Estimates (Log10 Scale)	*p*	Between-Subject Variability	Within-Subject Variability
Time on treatment (Weeks)	Intercept (Week 4)	0.159	0.02	27%	28%	0.145	0.51	22%	26%
	Week 8	0.166	0.02			0.151	0.03		
	Week 12	0.072	0.28			0.044	0.51		
	Week 24	0.014	0.83			−0.030	0.66		
	Week 48	0.155	0.02			0.115	0.09		
Baseline ALT	Intercept	0.100	0.24	27%	28%				
	ALT	0.000	0.18			0.004	0.03		
Baseline ALP	Intercept	−0.070	0.48	24%	28%				
	ALP	0.000	0.01			0.001	0.008		
Baseline Total Cholesterol	Intercept	0.310	0.01	27%	27%				
	Total Cholesterol	0.000	0.19			0.003	0.03		
Baseline LDL	Intercept	0.390	0.00	25%	28%				
	LDL	0.000	0.01			−0.005	0.03		
Baseline Triglycerides	Intercept	0.360	0.01	27%	28%				
	Triglycerides	0.000	0.07			−0.003	0.02		
*ABCB1 c.4036A>G*	Reference (A/A)	0.100	0.16	27%	27%				
	A/G or G/G	0.150	0.15			0.011	0.91		
*SLCO1B1* g.38664C>T	Reference (C/C)	0.310	0.00	25%	28%				
	*C/T* or *T/T*	−0.220	0.04			−0.205	0.09		
SLCO1B1 *1B	Reference (*A/A* or *A/G*)	0.230	0.00	27%	27%				
	*G/G*	−0.190	0.06			0.048	0.70		
Baseline AST	Intercept	0.120	0.08	26%	28%				
	AST	0.220	0.08						

* Only predictor variables with a *p*-value ≤ 2 from the univariate analysis are listed. ALP: alkaline phosphatase; ALT: alanine aminotransferase, AST: aspartate transaminase, LDL: low-density lipoprotein.

## Data Availability

All data accessed and analyzed in this study are available in the article.

## Supplementary Materials

The following are available online at https://www.mdpi.com/article/10.3390/jpm11121303/s1, Table S1: Effect of genotype on plasma efavirenz concentration at the 4th, 8th 12th, 24th, and 48th weeks of antiretroviral treatment among HIV-infected children using one-way ANOVA.
