# Supplementary material for: Predictors of Efavirenz Plasma Exposure, Auto-Induction Profile, and Effect of Pharmacogenetic Variations among HIV-Infected Children in Ethiopia: A Prospective Cohort Study
Source: J Pers Med. 2021 Dec 5;11(12):1303. doi: 10.3390/jpm11121303 (PMC8707067; doi:10.3390/jpm11121303)
Supplement: Supplementary file 1 [file jpm-11-01303-s001.zip › jpm-1439178-supplementary.pdf]

Supplementary Table 1. Effect of genotype on plasma efavirenz concentration at the 4th, 8th 12th, 24th and 48th weeks of antiretroviral treatment among HIV infected children using one-way ANOVA.

Genotype		n	Week 4			Week 8			Week 12			Week 24			Week 48		
			Geometric mean	Standard Error	*P*	Geometric mean	Standard Error	*p*	Geometric mean	Standard Error	*p*	Geometric mean	Standard Error	*p*	Geometric mean	Standard Error	*p*
CYP2B6*6	*1/*1	48	1.787	1.12	< 0.001	2.265	1.13	0.002	1.760	1.15	0.002	1.663	1.13	<0.001	2.196	1.13	<0,001
	*1/*6	44	2.505	1.14		2.095	1.19		2.281	1.24		2.648	1.13		1.810	1.22	
	*6/*6	9	6.183	1.28		8.193	1.43		8.715	1.64		11.219	1.52		8.361	1.22	
CYP3A5	*1/*1	6	2.253	1.20	0.98	2.628	1.09	0.64	0.827	1.53	0.23	1.537	1.43	0.28	1.008	1.57	0.07
	*1/ *3 or *1/*6	34	2.308	1.24		2.322	1.27		2.129	1.39		3.048	1.29		3.754	1.22	
	*3/*3 or *3/*6	61	2.373	1.11		2.513	1.15		2.532	1.12		2.315	1.11		1.894	1.16	
**UGT2B7 rs372 G>A**	*A/A*	17	2.336	1.21	0.38	2.398	1.39	0.59	3.237	1.33	0.43	2.416	1.42	0.91	2.970	1.30	0.45
	A/G	57	2.582	1.12		2.259	1.17		2.321	1.19		2.564	1.15		2.029	1.19	
	G/G	27	1.920	1.23		2.957	1.18		1.878	1.30		2.309	1.18		2.646	1.22	
ABCB1 c.3435C>T	*C/C*	65	1.970	1.12	0.004	2.173	1.15	0.09	1.877	1.16	0.03	2.187	1.11	0.05	2.342	1.12	0.77
	T carriers	36	3.468	1.15		3.190	1.22		3.821	1.26		3.334	1.29		2.220	1.34	
ABCB1 *c.4036A>G*	*A/A*	70	2.241	1.11	0.49	2.308	1.15	0.44	2.073	1.18	0.44	2.508	1.13	0.84	2.246	1.16	0.76
	G carriers	31	2.566	1.20		2.786	1.21		2.798	1.22		2.402	1.23		2.416	1.23	
SLCO1B1 *1B	*A/A*	11	2.883	1.49	0.5	2.088	1.43	0.89	2.328	1.39	0.12	2.934	1.35	0.54	3.006	1.35	0.78
	A/G	50	2.541	1.13		2.553	1.22		3.002	1.20		2.708	1.17		2.234	1.22	
	G/G	40	2.087	1.16		2.439	1.14		1.697	1.23		2.141	1.17		2.256	1.16	
SLCO1B1 *5	**1/*1*	76	2.230	1.12	0.57	2.831	1.13	0.14	2.277	1.19	0.99	2.679	1.15	0.42	2.394	1.16	0.65
	**1/*5*	22	2.775	1.17		1.715	1.32		2.344	1.17		2.099	1.14		1.998	1.25	
	**5/*5*	3	1.968	1.07		1.635	1.27		2.178	1.55		1.432	1.96		3.583	1.09	
	C/C	25	2.617	1.20	0.76	2.000	1.33	0.49	2.525	1.19	0.43	2.445	1.17	0.65	2.204	1.23	0.85
	C/T	47	2.211	1.15		2.790	1.17		2.563	1.26		2.708	1.17		2.211	1.24	

**SLCO1B 1 g.38664 C>T**	*T/T*	29	2.325	1.19	2.448	1.18	1.741	1.26	2.144	1.26	2.566	1.18
